# Replicable Interprofessional Competency Outcomes from High-Volume, Inter-Institutional, Interprofessional Simulation

**DOI:** 10.3390/pharmacy4040034

**Published:** 2016-10-25

**Authors:** Deborah Bambini, Matthew Emery, Margaret de Voest, Lisa Meny, Michael J. Shoemaker

**Affiliations:** 1 Kirkhof College of Nursing, Grand Valley State University, 301 Michigan St NE, Grand Rapids, MI 49503, USA; bambinid@gvsu.edu; 2 College of Medicine, Michigan State University, 15 Michigan St NE, Grand Rapids, MI 49503, USA; mathew.emery@hc.msu.edu; 3 College of Pharmacy, Ferris State University, 25 Michigan St NE, Grand Rapids, MI 49503, USA; lisameny@ferris.edu; 4 Department of Physical Therapy, Grand Valley State University, 301 Michigan St NE, Grand Rapids, MI 49503, USA; shoemami@gvsu.edu

**Keywords:** interprofessional education, simulation, team-based care, interprofessional curricula

## Abstract

There are significant limitations among the few prior studies that have examined the development and implementation of interprofessional education (IPE) experiences to accommodate a high volume of students from several disciplines and from different institutions. The present study addressed these gaps by seeking to determine the extent to which a single, large, inter-institutional, and IPE simulation event improves student perceptions of the importance and relevance of IPE and simulation as a learning modality, whether there is a difference in students’ perceptions among disciplines, and whether the results are reproducible. A total of 290 medical, nursing, pharmacy, and physical therapy students participated in one of two large, inter-institutional, IPE simulation events. Measurements included student perceptions about their simulation experience using the Attitude Towards Teamwork in Training Undergoing Designed Educational Simulation (ATTITUDES) Questionnaire and open-ended questions related to teamwork and communication. Results demonstrated a statistically significant improvement across all ATTITUDES subscales, while time management, role confusion, collaboration, and mutual support emerged as significant themes. Results of the present study indicate that a single IPE simulation event can reproducibly result in significant and educationally meaningful improvements in student perceptions towards teamwork, IPE, and simulation as a learning modality.

## 1. Introduction

The role of interprofessional education (IPE) experiences for students from all health professions has never been more important. While it has been 16 years since the Institute of Medicine published the hallmark report To Err is Human [1], deaths from errors have continued to rise. During this same timeframe, growing costs and resource constraints have further emphasized the need to improve health care delivery. A rapidly growing body of literature documents the positive impact of inter-professional team based care on both the quality and cost of care [2]. However, there is a gap between the education of health professionals and the actual practice of healthcare [3]. Educators recognize the need to stop educating in silos and build more interprofessional experiences into the various curricula. Discipline-specific accreditation agencies have added IPE to their requirements, but many barriers remain [4]. Time constraints, scheduling, and accessibility are among the most common barriers [5,6]. For some programs, availability of other health professional education programs is lacking [7,8]. Furthermore, IPE simulation experiences should incorporate realistic, authentic scenarios, have team-based structured debriefing, and promote collaborative, interprofessional teamwork and communication [9], which can be challenging with large IPE simulation events.

There is limited prior research regarding IPE experiences accommodating a high volume of students (>100) from several disciplines and from different institutions. Bridges et al. [6] report on the collaboration between three universities and their interprofessional education programs. They describe didactic, community-based experiential, and simulation-based approaches to IPE that are able to accommodate hundreds of students each year. In each of the approaches there was formative and summative evaluation, but they did not report on any formal assessment of outcomes, either quantitatively or qualitatively.

Buckley et al. [10] described a half-day interprofessional simulation using three different scenarios with different combinations of five health care disciplines for a total of 190 students. They reported favorable quantitative and qualitative changes in student perceptions of IPE, but did not use a validated questionnaire with known psychometric properties.

Vouri et al. [11] outlined the successful negotiation of the challenges associated with interprofessional, inter-institutional collaboration between a private college of pharmacy with three neighboring schools to construct a required IPE seminar course, however, no empirical data on outcomes were analyzed or reported.

Finally, King et al. [7] described an experience involving 78 students from multiple disciplines from four institutions. They utilized two interprofessional student cohorts, with one assigned to a mannequin-based simulation and the other to a standardized patient (SP)-based simulation. The primary assessment measure was change in communication and teamwork using a pre/post assessment with the West England Questionnaire tool. Overall student perceptions of their communication and teamwork improved after participating in the experience. The authors noted a significantly greater improvement in those students who participated in the SP-based simulation. However, no analysis of effect size was noted for this or any of the other results of the study, nor was there a comparison of the effect of the simulation on the individual professions.

In considering the aforementioned weaknesses of the existing literature, especially with regard to the number of students, type and level of measurement, analyses utilized, and reproducibility of results, further research on high-volume, inter-institutional IPE activities is clearly warranted. Therefore, the present study sought to answer the follow research questions: (1) To what extent does a single, large, inter-institutional, and IPE simulation event improve student perceptions of the importance and relevance of IPE and simulation as a learning modality? (2) Is there a difference in student perceptions of the importance and relevance of IPE based on discipline prior to an IPE event and does an IPE simulation impact all disciplines equally? (3) Are any effects of the IPE simulation on the importance and relevance of IPE and simulation reproducible?

## 2. Materials and Methods

During the spring semester of 2014, 180 nursing, medical, and pharmacy students from the three different universities took part in an IPE simulation event. Student and faculty responses to this event provided impetus to repeat, refine, and prospectively study the event. The experience was repeated with a different student cohort (*n* = 144) during the Fall 2014 semester and again with a third student cohort (*n* = 146) in Spring 2015, with the addition of a fourth discipline, physical therapy. This article describes the learning experiences and outcomes of the Fall 2014 and the Spring 2015 simulation-based interprofessional education experiences. Specifically, student perceptions of the importance and relevance of IPE and simulation were assessed quantitatively and qualitatively. All subjects gave their informed consent for inclusion before they participated in the study. The study was conducted in accordance with the Declaration of Helsinki, and the protocol was approved by the Ethics Committee of Grand Valley State University (672729-2) and Ferris State University (141104).

### 2.1. Participants

All nursing students in the second to last semester of a Bachelor’s of Science in Nursing, prelicensure program, third-year Doctor of Medicine students in the core competency clinical skills courses, and third-year Doctor of Pharmacy students were required to participate in the *Towards Collaborative Practice* (TCP) simulation. During the April 2015 event, third-year Doctor of Physical Therapy students were also included.

### 2.2. Simulation Planning and Preparation

Students were sent an email describing the event, detailing logistical issues such as location of event, and assignments to complete prior to the simulation experience. On the day of the experience, all students were asked to complete the Attitude Towards Teamwork in Training Undergoing Designed Educational Simulation (ATTITUDES) Questionnaire [12] prior to receiving an orientation and prebrief on the day of the event. Students were also asked to consent to the dissemination of aggregate findings from the experience to educational audiences.

Students who consented were placed in small patient care teams of 4–7 students that included 1–2 students from each of the healthcare professions. Students who did not consent to have their data included in the analysis were grouped together to complete the activity. All students, regardless of consent, completed the same simulated patient care experience.

Student teams were assigned to a standardized patient, executed a simulated patient care experience and worked together to create a plan of care, as well as complete discipline-specific clinical documentation. Following the simulation, all students participated in a debriefing of the simulation, led by faculty.

### 2.3. Simulation Logistics

Event duration for each participant required 3.5 h to complete, in addition to 30–60 min of preparation prior to arrival at the simulation center as outlined in Table 1. Following completion of baseline measurements and event prebriefing, 90 min were provided for the actual simulation. Teams were given the opportunity to decide how they wanted to organize and distribute their time with the patient. Following completion of the simulation and the group debriefing, students completed the post-simulation questionnaires.

### 2.4. Simulated Cases

The simulated encounters used for all events consisted of standardized patient cases. All cases were written to be gender neutral, allowing them to be portrayed by either a male or female. In addition, all cases were designed to create challenges that would require collaboration and communication between the disciplines. Content validity was sought through expert opinion of the faculty representing each discipline.

Two standardized patient cases, given the last names Dalton and O’Conner, were used for the Fall 2014. Half of the learners were assigned to the “Dalton” case and half assigned to the “O’Conner” case. Two cases were used to minimize student sharing of case information for those students participating in the event later in the day.

Dalton was a 58-year-old with multiple medical problems, including hypertension and diabetes, who was sent to the emergency department (ED) for follow-up of abnormal outpatient labs, including an elevated blood glucose. The labs had been ordered after the patient was seen earlier in the day by the primary care physician (PCP) for a “bad cold”. In addition to addressing the patient’s elevated blood glucose, learners discover the need to clarify the patient’s current medication regimen as there are discrepancies between the medications as reported by the patient, a hand-written list brought by the patient, and a brown paper bag containing numerous medication bottles. If asked, the patient discloses that he/she stopped taking the insulin due to poor appetite. With careful questioning, the SP discloses that his/her spouse, who is currently out-of-town, does most of the cooking and also lays out the patient’s medication on a daily basis. Further testing in the ED also reveals mild hyponatremia. Key challenges for this case included clarifying and then adjusting the patient’s medication regimen, addressing the abnormal lab values, as well as identifying and addressing social stressors.

O’Connor was a recently widowed 75 year-old brought to the ED by his/her adult child who voiced concern about recent change in mental status and a complicated medication regimen. Key challenges to be identified and addressed by the team included identification of dangerous medication interaction associated with the use of an herbal supplement (du hou) for arthritis pain, development of an effective pain management strategy, differential diagnosis of depressive and cognitive changes, and development of an appropriate strategy for helping the patient better cope with the loss of his/her spouse.

A single case, “Morgan South” was created for the Spring 2015 event in order to better incorporate the physical therapy students. Experience from the Fall 2014 event showed that a second case was not necessary. Morgan South was an anticoagulated 72 year-old with a history of intermittent atrial fibrillation who was directly admitted to the ED from the primary care office due to UTI with associated progressive weakness and falls. Key challenges to be addressed by the team included the assessment of fall risk, appropriateness for anticoagulation, potential interactions between warfarin and antibiotic therapies, evaluation of orthostatic hypotension, and various medication reconciliation issues including use of ibuprofen concurrent with warfarin.

### 2.5. Outcome Measures

Effects of the simulation on attitudes towards teamwork, IPE, and simulation as a learning modality were measured using the ATTITUDES Questionnaire [11]. The subscales of the ATTITUDES Questionnaire [11] were developed using factor analysis in a sample of health professional students and demonstrated high internal reliability overall (α = 0.95) and within each subscale (α = 0.78 to 0.91). Higher scores indicate more positive attitudes. Written responses to open-ended questions were also utilized to further explore themes related to teamwork and IPE as well as organization and execution of the simulation activity that would otherwise not emerge through discrete, Likert scale-based questions.

### 2.6. Data Analysis

The change in ATTITUDES Questionnaire overall score and subscale scores were analyzed using dependent *t*-tests to compare attitudes regarding IPE and practices before and after the learning experience. Effect sizes for changes in overall score and each subscale were calculated using Cohen’s *d*. One-way ANOVA was used to examine between-discipline differences for baseline and change in ATTITUDES Questionnaire overall score and subscale scores. Independent *t*-tests were used to examine differences between overall and discipline-specific Fall and Spring event cohorts to determine whether the outcomes were replicable. Responses to open-ended questions were analyzed independently by two blinded research assistants for common themes. Collaborative analysis of this data into themes was completed as described by Miles and Huberman [13] where the comments were manually coded by underlying key terms, key phrases, creating clusters, and identifying themes.

## 3. Results

The basic demographics of the participants are outlined in Table 2. Over 90% of each cohort consented to participation in the research component of the event.

With regard to the impact of the simulation on student perceptions towards teamwork, IPE, and simulation as a learning modality, there was a statistically significant improvement in ATTITUDES Questionnaire scores from 130.0 (11.9) to 138.0 (13.4) points (*t* = 11.59, *p* < 0.001). This overall mean change of 8 points represented a medium effect size of 0.69 and exceeds the minimum detectable change (MDC) of 6.88 points calculated by the present authors based on the test–retest reliability coefficient reported by Sigalet et al. [11]. As presented in Table 3, there was a statistically significant improvement across all ATTITUDES subscales with medium effect sizes observed in all but the Communication subscale.

ATTITUDES did not improve to the same extent across each discipline, with the medical students demonstrating a significantly lower amount of change [4.7 (15.1) points, F = 2.98, *p* = 0.032] compared to the 8.9–9.9 point change in the other disciplines; this was primarily due to smaller changes in the Communication (0 points) and Situation Awareness (0.76 points) subscales. It should be noted that the standard deviation of change (15.1 points) for the medical students was over three times greater than the mean change of 4.7 points, indicating substantial variability in student response to the simulation. It is also important to note that the medical students, at baseline, had significantly lower overall and subscale scores except for the Communication subscale.

The aforementioned results were replicable with both cohorts demonstrating similar ATTITUDES improvements in overall (*t* = −0.087, *p* = 0.931) and subscale scores (*t* = −1.1 to 0.41, *p* = 0.28 to 0.95). Additionally, the aforementioned differences in effect based on discipline were consistent in both cohorts.

With regard to qualitative analysis of the open-ended questions (Table 4), several important and relevant themes emerged for each question. Time management issues and being unsure of the roles and overlap of other professions were self-identified by participants as barriers to collaboration on developing a plan of care for the simulated patient. Teamwork and mutual support, as well as gaining perspective on effective interprofessional teamwork were perceived as some of the important benefits of working closely together on the care of the simulated patient. Of the 161 responses as to whether the IPE simulation experience should be incorporated into the curriculum, only 8.7 percent (*n* = 14) commented that the activity was too artificial or was too repetitious of experiences encountered during clinical rotations. Finally, suggestions for further improving the simulation experience from 31% of participants included a desire for more communication and instruction from the facilitators about the objectives, logistics, and flow of the activity.

## 4. Discussion

Patient outcome and program accreditation standards have established IPE as a critical component of health professional education, challenging programs to find innovative and efficient ways to overcome the numerous barriers to IPE. The present study sought to address gaps in the literature with regard the use and effectiveness of simulation as an IPE learning modality for a high volume of students across multiple institutions. Our results indicate that a single IPE simulation event can reproducibly result in significant and educationally meaningful improvements in student perceptions towards teamwork, IPE, and simulation as a learning modality, which adds to the existing knowledge in several important ways.

First, unlike previous studies investigating high-volume and/or interinstitutional IPE, we quantified the effects of the IPE activity using an established, validated questionnaire allowing for estimation of effect size and how the magnitude of change compared to the MDC value. In the present study, improvements in the ATTITUDES overall scores exceeded the MDC value to 6.88 points. The 2012 study by Buckley et al. [9] is closest in scope and design to the present study, and although they observed improvements in student perceptions of IPE, they did not use an established measurement tool.

The second unique addition of the present study to existing literature is the specific analysis of whether the IPE learning effects were reproducible with a different cohort using a different case and discipline combination. Bridges et al. [6] studied various interinstitutional IPE activities, but did not report on specific effects or how those effects compared between the various activities and between the same activities in different years. Buckley et al. [6] did not specifically report on the reproducibility of the results among their varying simulation scenario and discipline combinations. In the present study, there was no difference in outcome between those learners who were provided with the experience in the Fall 2014 semester as compared to those in the Spring 2015 semester. This highlights two important concepts. Firstly, the case must be designed to include challenges for each disciplined involved. The addition of physical therapy students required that the case contain problems that could be uniquely addressed by the physical therapist and that required communication and collaboration with other disciplines. When done properly, including an additional discipline in an IPE simulation does not appear to detract from the outcomes for all learners included in the event.

The third unique addition of the present study to existing literature is the consideration of the effect of the simulation event on different learner levels. No prior study has clearly commented on how timing of IPE simulation within a discipline’s respective curriculum may affect the learning outcome. The medical, nursing, and pharmacy students were at different points in their professional education in each of the events, and there was no difference in outcome. Although King et al. [7] observed that communication and teamwork scores “were significantly higher with students in year 2 than in year 4”, their sample size precluded controlling for the confounding effect on institution. Of course, simulation planners need to take into account whether participants will have had a basic set of knowledge and skills required to evaluate and manage the clinical problems portrayed in a given simulation, but precise timing in the curriculum may not be important for achieving IPE objectives.

There may however be important issues related to learner level between disciplines. Similar to the results of Buckley et al. [9] with regard to differences in how each profession responded to the IPE simulation, the magnitude of change across all disciplines was dissimilar, with medicine demonstrating a lower amount of change in overall and subscale scores, especially communication. It should be noted that there was substantial variability in the baseline and change scores for the medical students, with standard deviations of change in overall and subscale scores at least three times greater the mean amount of change. We initially hypothesized that this variability in response is due, in part, to greater variability in prior experience with interprofessional and team-based experiences. Indeed, the average age of the medical students was nearly 2–3 years older than learners from other disciplines. However, this does not account for the lower baseline scores. Another possible explanation is that of learner level. At the time of the simulation, the medical students were already engaged in full-time clinical rotations. It may be that some of these students did not value a simulated event by that stage in their professional education. The effect of prior clinical and life experiences on response to IPE simulation and the implementation of strategies to ensure equal magnitude of benefit for all learners is an area ripe for future investigation, however inclusion of learners with a diverse range of prior experiences is perhaps a critical ingredient for the success of IPE.

A strength of this study is the inclusion of both quantitative and qualitative analysis of learners’ perceptions of IPE. The inclusion of student comments allows for a more robust understanding of the learners’ thoughts and feelings related to the IPE experience. A majority of learners felt that events such as these should be routinely implemented in the curriculum. This is consistent with the increased emphasis on IPE within the curriculum by the accrediting bodies of the various health professions.

Qualitative analysis of learner perceptions was also helpful in revealing important elements of simulation logistics, especially communication between instructors and learners. Learners reported that they would like more clear and comprehensive communication and direction regarding the logistics and expectations of the experience. Although over time we have continued to refine the materials and information provided to learners prior to the IPE simulation, further improvement is needed. Since completion of the study, we have placed additional emphasis on the prebriefing that the students receive upon arrival, and provide supporting materials well in advanced of the event. Future studies are needed to determine the key communication elements that must be provided to learners to best position them for success. Additionally, feedback regarding student performance is another area in need of improvement. The optimal methods for student assessment and provision of feedback requires further study.

A primary limitation of the present study is it reliance on learner perception and self-report. The impact of one or more IPE simulations on objective observation of team dynamics through video analysis and evaluation of health care team decisions and their impact on the quality of patient care would be particularly enlightening as to the effectiveness of IPE simulation in a clinically relevant context.

## 5. Conclusions

Results of the present study indicate that a single IPE simulation event can reproducibly result in significant and educationally meaningful improvements in student perceptions towards teamwork, IPE, and simulation as a learning modality. Three unique contributions of these results to existing literature are the use of a valid survey instrument, the assessment of replicability, and considerations of responses among different disciplines and learner levels.

## Figures and Tables

**Table 1:** Simulation Logistics.

Component	Time	Details
Preparation	60 min	Students are emailed details regarding preparatory work (e.g., articles to read), time/location of the event, and event outline.
Arrival	30 min	Students arrive at the prebriefing room.
Prebriefing		Welcome. ATTITUDES Questionnaire. Discussion of objectives and explanation of logistics.
Simulation	90 min	Patient case. Students assess the patient using discipline-specific knowledge, skills, and attitudes and collaborate to develop a plan of care. Students complete discipline-specific assignments (e.g., documentation of assessments, medication reconciliation, medical disposition and plan). Students discuss plan of care with patient.
Team Debriefing	30 min	Discussion about case and team dynamics with instructor.
Large Group Debriefing	45 min	Debrief with focus on teamwork with faculty team.
Conclusion	15 min	Survey of Team Dynamics, ATTITUDES Questionnaire. Summary Evaluation.

**Table 2:** Participant Demographics.

	N	Age [Mean (Std. Dev.)]	Gender (% Female)
**Overall**	290	24.4 (3.8)	67.3
Cohort 1	144	24.3 (4.6)	73.5
Cohort 2	146	24.5 (2.9)	66.7
**Medicine**	72	26.5 (3.2)	43.1
Cohort 1	40	26.1 (2.6)	40
Cohort 2	32	27.1 (3.8)	46.9
**Nursing**	127	22.9 (4.0)	88.9
Cohort 1	72	23.1 (5.0)	93
Cohort 2	55	22.7 (2.2)	83.6
**Pharmacy**	59	24.5 (3.7)	66.1
Cohort 1	36	24.8 (4.6)	72.2
Cohort 2	23	24.1 (1.4)	56.5
**Physical Therapy**	34	25.1 (1.0)	63.6
Cohort 1	-	-	-
Cohort 2	34	25.1 (1.0)	63.6

**Table 3:** Change in ATTITUDES Subscales (Both Cohorts).

	Baseline	Change
**Overall**	130.0 (11.9)	8.1 (11.6) *
Medicine	125.4 (15.0) #	4.7 (15.1) *#
Nursing	131.8 (9.8)	8.9 (9.7) *
Pharmacy	130.7 (12.0)	9.9 (10.0) *
Physical Therapy	130.8 (9.8)	9.9 (10.9) *
**Relevance of IPE**	30.2 (3.5)	2.0 (3.0) *
Medicine	28.7 (4.4) #	1.3 (3.7) *
Nursing	30.9 (2.9)	2.2 (2.6) *
Pharmacy	30.5 (3.5)	2.6 (2.8) *
Physical Therapy	30.8 (2.7)	1.9 (3.2) *
**Relevance of Simulation**	21.1 (2.5)	1.7 (2.6) *
Medicine	19.9 (3.1) #	1.4 (3.1) *
Nursing	21.4 (2.0)	2.0 (2.3) *
Pharmacy	21.5 (2.6)	1.8 (2.3) *
Physical Therapy	21.8 (2.0)	1.6 (2.5) *
**Communication**	35.7 (3.2)	1.4 (3.4) *
Medicine	35.3 (3.9)	0.0 (4.2) #
Nursing	36.1 (2.8)	1.6 (2.8) *
Pharmacy	35.3 (3.1)	2.0 (2.9) *
Physical Therapy	35.6 (2.8)	2.0 (3.1) *
**Situation Awareness**	17.2 (1.7)	1.2 (1.9) *
Medicine	16.8 (2.0) #	0.8 (2.0) *#
Nursing	17.5 (1.5)	1.2 (1.6) *
Pharmacy	17.3 (2.1)	1.5 (2.1) *
Physical Therapy	17.2 (1.6)	1.7 (1.8) *
**Roles and Responsibility**	25.6 (2.9)	1.9 (3.1) *
Medicine	24.6 (3.9) #	1.3 (4.1) *
Nursing	26.0 (2.4)	2.0 (2.7) *
Pharmacy	25.9 (2.5)	2.1 (2.6) *
Physical Therapy	25.4 (2.4)	2.7 (2.5) *

* Paired *t*-tests difference *p* < 0.05; # One-Way ANOVA difference between Groups difference from other professions using planned contrasts *p* < 0.05.

**Table 4:** Results of Qualitative Analysis of Open-Ended Questions.

Question	Themes	% Students Contributing to Theme
What barriers did you encounter as you collaborated with other professional students to develop a plan for your patient?	Time Management	35.0%
	Unsure of roles of other professions/overlap of professions	32.4%
What are some of the benefits of partnering with other health professionals?	Collaboration/Mutual Respect	57.9%
	Gaining perspective on effective collaboration	51.0%
Do you think there is value to further incorporate this experience into the curriculum?	Great experience/fun/good to work with other providers/helpful-should do it again	51.0%
What would you suggest to enhance/improve the experience for the next group of students?	No change	42.4%
	More communication/direction/goals from instructors and facilitators	31.4%
